# Ancestral Haplotype Retention and Population Expansion Determine the Complicated Population Genetic Structure of the Hilly Lineage of *Neolucanus swinhoei* Complex (Coleoptera, Lucanidae) on the Subtropical Taiwan Island

**DOI:** 10.3390/insects12030227

**Published:** 2021-03-05

**Authors:** Cheng-Lung Tsai, Kôhei Kubota, Hong-Thai Pham, Wen-Bin Yeh

**Affiliations:** 1 Department of Entomology, National Chung Hsing University, Taichung City 40227, Taiwan; cltsai.lucanid@gmail.com; 2 Department of Forest Science, Graduate School of Agricultural and Life Sciences, The University of Tokyo, Tokyo 113-8657, Japan; kohei@fr.a.u-tokyo.ac.jp; 3 Ft. Lauderdale Research & Education Center, Department of Entomology and Nematology, University of Florida, Ft. Lauderdale, FL 33314, USA; 4 Mientrung Institute for Scientific Research, Vietnam Academy of Science and Technology, Hue 49000, Vietnam; phamthai1976@yahoo.com; 5 Vietnam National Museum of Nature & Graduate School of Science and Technology, Vietnam Academy of Science and Technology, Hanoi 10000, Vietnam

**Keywords:** *Neolucanus swinhoei* complex (NSC), ancestral haplotype determination, mountain hindrance, dispersal route, recent population expansion

## Abstract

**Simple Summary:**

*Neolucanus swinhoei* is a widespread species of stag beetle distributed across the subtropical Taiwan Island. The biological features of weak flying capability, two-week adult lifespan, and strong dependence on forests are the key factors determining its genetic differentiation. We investigated the influence of historical climatic oscillations in Pleistocene on the distribution of *N*. *swinhoei*. The complex population genetic structure and dispersal routes of eastern populations are affected by the geographic isolation of the north/south-oriented Central Mountain Range (CMR) during periodical Pleistocene glaciations. Four possible shelters were inferred for the distribution of *N*. *swinhoei* in each geographic region during the periodical glaciations. The coexistence of widely distributed ancestral haplotypes and the locally derived ones are deduced to be the possible explanation of long-distance dispersal events for *N*. *swinhoei*. In the presence of the CMR, the northern and southern low mountains are proposed as alternative routes for *N*. *swinhoei*. The present study proposes dispersal routes from eastern to western Taiwan, in contrast to the more common scenarios of western-to-eastern dispersal found in other studies.

**Abstract:**

The present study demonstrates that the complicated genetic structure of the hilly lineage of the *Neolucanus swinhoei* complex was driven by its biological features and habitat requirements as well as hindrance by the CMR during periodical Pleistocene glaciations. The results revealed a tendency of geographical differentiation and major and sub- lineage divergences before and after the Riss glaciation, followed by stable population growth during Würm glaciation. At least four refugia were inferred for *N*. *swinhoei* during the Riss–Würm glaciations. The ancestral haplotype retention in the cytochrome oxidase subunit I (COI) gene and compensated substitution in 16S rRNA gene is a possible evolutionary scenario resulting in the inconsistent evolution pattern between COI and 16S rRNA gene coupled with the long-distance dispersal of *N*. *swinhoei*. Although the CMR did hinder the dispersal of *N*. *swinhoei*, its ancestors may have dispersed to eastern Taiwan through the northern and southern low mountains of the CMR before the Riss glaciation. Our finding suggests that the population growth in the Würm glaciation led a dispersal back to western Taiwan, which is contrast to the more common dispersal scenario from western Taiwan to eastern populations proposed in other studies.

## 1. Introduction

Historical climatic fluctuations during the Pliocene‒Pleistocene period have been considered a significant factor affecting species diversification and population dynamics of extant organisms [1,2,3]. In the low-latitudinal Amazonian and Andean regions where climates are more stable, speciation events occurred mainly during Pliocene glaciation [1,2,4,5,6,7]. However, relevant studies in Europe and North America have revealed that temperate species were significantly affected by successive ice sheets driven by the 100,000-year Pleistocene glacial cycles [2,3,8]. In the last decade, a congruent scenario based on the comparative phylogeography has revealed that several divergent lineages could be found within a species. The closely related plants and animals studied in China, Japan, Madagascar, and New Zealand have revealed that the divergent pattern predominantly occurred during Pleistocene glaciations [9,10,11,12]. Whether the timing of diversification of each lineage was affected by the same glacial event requires further investigation.

The Island of Taiwan, situated majorly in a subtropical region, emerged above sea level approximately 5–6 Mya due to collisions between the Philippine Sea plate and the Eurasian plate [13,14]. The intense uplift approximately 3–1 Mya [13,14] resulted in the emergence of the Central Mountain Range (CMR), which stretches north–south in middle of the island. Together with the Syueshan Mountain Range (SMR) and Yushan Mountain Range, these mountains have over 250 peaks higher than 3000 m. The geographical distribution and dispersal routes of organisms in Taiwan were significantly affected by the mountainous topography in the past one million years. Therefore, the CMR has been considered an important geographical barrier playing a role in speciation and east–west genetic differentiation of organisms in Taiwan [15,16,17,18,19,20,21,22]. Despite the nearly 100 studies published on genetic differentiation, the dispersal routes to eastern Taiwan remain unclear. The hills and lower mountains in the northern and southern parts of the CMR are known to be associated with the dispersal routes of eastern populations. For examples, the common cricket *Loxoblemmus appendicularis* shifted to eastern Taiwan through a southern route [21] and the rhacophorid tree frog *Kurixalus eiffingeri* via a northern route [23].

The *Neolucanus swinhoei* complex (NSC), a group of diurnal stag beetles endemic to Taiwan, are characterized by their complicated phylogeographical history due to the influence of the CMR and periodical Pleistocene glaciations [16]. Two major lineages, namely montane and hilly, comprised nearly 90% of specimens from western Taiwan [16]. Calibration dating revealed that the diversification of these lineages occurred during the mid-Pleistocene (0.59–0.75 Mya). The hilly lineage diversified approximately 0.37 Mya after the Mindel glaciation and subsequently underwent an expansion during the Last Glacial Maximum (LGM). Thereafter, complex differentiation processes occurred between montane and hilly lineages under Pleistocene glacial cycles in the last 300,000 years [16]. Another survey by Tsai and Yeh [20] reported that the hilly lineage was also present in eastern Taiwan. The population genetic differentiation of the hilly lineage of NSC should therefore be explored in greater detail.

Determining the population genetic structure and historical dispersal of organisms driven by periodical Pleistocene glaciation and geographical barriers in the Island of Taiwan has always been a challenge [16,21,24]. The biological features of *N*. *swinhoei*, including weak flight capability, a 2-week adult lifespan, and an altitudinal distribution limited to <1500 m, may shape its differentiation pattern under CMR hindrance [16]. The strong dependence of this species on native forests for larval diet and habitat may also be responsible for their diversification within each mountain area [16,25]. Therefore, we suggest that genetic differentiation of *N*. *swinhoei* may be closely related with the recurrent climatic oscillations and CMR hindrance during the Pleistocene.

We propose a hypothesis involving refugia isolation during the Riss glaciation and secondary contact triggered by the Würm glaciation, namely LGM, for the hilly lineage of NSC, which has been demonstrated in plants and other insects [20,26,27,28]. During glaciations, populations isolated in refugia may fix mutations by means of genetic drift. However, if population are not isolated for a sufficient amount of time, there may exist a coexistence of ancestral and newly derived haplotypes. When the interglacial period began, isolated populations appeared to have the opportunity for gene flows with other populations through secondary contact. Therefore, the population genetic structure of the hilly lineage of NSC involving ancestral haplotype maintenance and new haplotype mutations is important in the examination of the impact of periodical Pleistocene glaciations.

In *N*. *swinhoei*, repeated isolation in and expansion from refugia driven by the 100,000-year Pleistocene glacial cycle may have caused the evolution of a complicated population genetic structure. The coexistence of widespread haplotypes and geographically specific ones in each population provides evidence for refugia isolations and ancestral synapomorphy during glacial cycles. In the present study, the mitochondrial genes of cytochrome oxidase subunit I (COI) and 16S rRNA gene were used to investigate the differentiation history of the hilly lineage of NSC without the use of nuclear amplicons because of the genetic admixture proposed by Tsai et al. [16]. The aims of this study were as follows: (1) to address the phylogenetic relationships and population genetic structure of the hilly lineage, (2) to elucidate the refugia effects on NSC differentiation history under recurrent Pleistocene glaciations and mountain hindrance, and (3) to reveal the historical dispersal of the hilly lineage and the origin of eastern populations. We hypothesized that the deduction of ancestral haplotypes and the newly derived mutations suggest refugia effects evolved under CMR hindrance and climatic oscillations during Pleistocene glaciations.

## 2. Materials and Methods

### 2.1. Sample Collection

**Figure 1:**
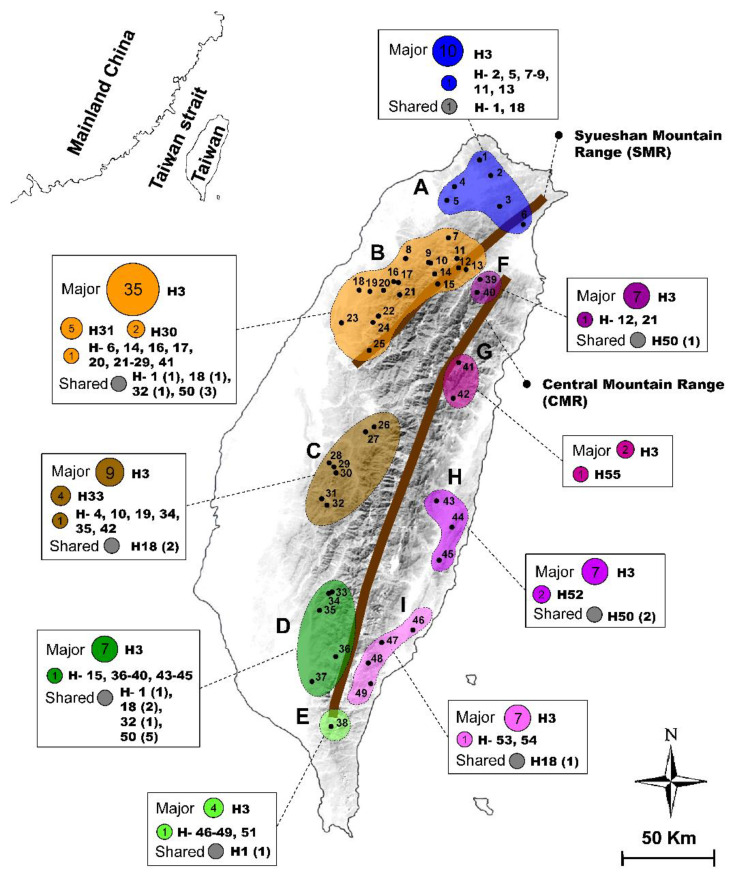
Collection localities of the hilly lineage of *Neolucanus swinhoei* complex (NSC). Nine populations were tentatively defined based on the topography of Taiwan Island. Haplotype compositions including major, unique, and some shared haplotypes are shown for each population. The denoted haplotype (H) numbers and individuals are shown based on the COI network. The geographic position of Central Mountain Range (CMR) and Syueshan Mountain Range (SMR) are labeled.

A total of 171 specimens of the hilly lineage of NSC were collected from 49 sites (Table 1, Figure 1) throughout Taiwan Island. These specimens included 70 newly collected stag beetles, 9 dried specimens of *N*. *swinhoei* from the Department of Plant Medicine, National Pingtung University of Science and Technology, Pingtung County, Taiwan, and 4 dried specimens from the National Museum of Natural Science, Taichung City, Taiwan. In addition, the COI and 16S rRNA gene sequences of 88 specimens published by Tsai et al. [16] and Tsai and Yeh [20] were retrieved from GenBank (Table S1). Among these samples, nine populations were tentatively defined based on the topography of major rivers and mountain ranges, for analyses of their population genetic structure and differentiation history. The congeneric species *Neolucanus nitidus* from China and Vietnam and individuals from the other three NSC lineages were used as outgroups (Table S1).

### 2.2. DNA Extraction, Amplification, and Sequencing

Genomic DNA was extracted from the metacoxa muscle using the QuickExtract DNA extraction kit (Epicentre Biotechnologies, Madison, WI, USA) and following the protocol of Tsai et al. [16]. The primer sets used to amplify COI and 16S rRNA genes were taken from Tsai et al. [16] and Yeh et al. [29] (Table 2). For dried specimens, CI–Neo–F was applied as the forward primer in the second run of a nested polymerase chain reaction (PCR). A PCR was conducted with 25 μL of the sample under the following programming conditions: initial denaturation at 94 °C for 2 min, 35 cycles at 94 °C for 40 s, 48–52 °C for 40 s, and 72 °C for 40 s, followed by final extension at 72 °C for 10 min. The PCR products were purified through treatment with shrimp alkaline phosphatase/exonuclease I (USB Products, Affymetrix, Cleveland, OH, USA) and then sequenced using an ABI 3730XL DNA Analyzer (Applied Biosystems, Foster City, CA, USA).

### 2.3. Phylogenetic Analyses

The timing of diversification for the hilly lineage of NSC was estimated in BEAST v. 1.6.1 [30] using a strict molecular clock algorithm. The congeneric species *N*. *nitidus* and specimens from the other three NSC lineages mentioned in Tsai et al. [16] were used as outgroups. The best-fit model for nucleotide substitution was determined in jModelTest 0.1 by using the Bayesian information criterion (BIC) [31]. The suitable model obtained in BEAST v. 1.6.1 for COI and 16S rRNA genes were TrN + I and TrN + I + G, respectively. Two sets of substitution rates were estimated for these stag beetles: (1) 1.77%/lineage per million years (Myr) and 0.53%/lineage/Myr for COI and 16S rRNA genes, calibration data from Aegean tenebrionid beetles [32]; (2) 1.15%/lineage/Myr and 1%/lineage/Myr for COI and 16S rRNA genes, rates used for general insects [33]. The Markov chain Monte Carlo (MCMC) analyses were run for 1 × 10^8^ generations with sampling every 1 × 10^4^ generations. The effective sample size (ESS) for the posterior distribution of estimated parameter values were analyzed in Tracer v. 1.5 [34] until the suggested value was reached; then, the initial 10% of the run was discarded as burn-in.

### 2.4. Genetic Structure and Haplotype Network Analyses

Analysis of molecular variance (AMOVA) was performed in Arlequin 3.1 [35] to estimate the genetic diversity indices containing haplotype diversity (h), nucleotide diversity (π), molecular variance, and neutrality tests. To detect the effects of isolation by distance on these stag beetles, the relationship between nucleotide divergences and geographical distances was analyzed using a linear regression analysis (Excel; Microsoft, Redmond, WA, USA). The pairwise genetic distances (p–distances) between individuals were calculated in MEGA 7.0 [36]. Geographical distances among populations were measured using Google Earth v. 6.1 (http://www.google.com/intl/zh-TW/earth/index.html (accessed on 1 October 2016)). 

Statistical parsimony network analysis was conducted in TCS v. 1.21 on the basis of a 95% connection limitation [37], and the alignment indel was considered the fifth state in 16S rRNA gene. In the COI network, the serial numbers of haplotypes were labeled according to the population order from A to I. The definition of ancestral haplotypes was crucial in depicting the differentiation history of the hilly lineage of NSC; however, the detection of ancestral haplotypes was challenging. Certainly, the major haplotype was more ancestral than the tip-extended haplotypes. The present study thus defined the major haplotype to be more ancestral than its tip-extended ones, which was considered the standard for relevant analyses and hypothesis testing. Moreover, the DELTRAN optimization in parsimony network was applied to define the evolved processes of ancestral and derived haplotypes. Therefore, the most recent mutation would take place as far toward the tips of the tree as possible.

### 2.5. Demographic History

The coalescent-based Extended Bayesian Skyline Plot (EBSP) based on COI + 16S rRNA genes was implemented to reconstruct historical population demographics for the hilly lineage using BEAST v. 1.6.1 [29]. The fittest nucleotide substitution models as determined in jModelTest 0.1 using BIC [30] for COI and 16S rRNA genes were HKY + G and HKY + I, respectively. The MCMC analyses were run for 1 × 10^8^ generations with sampling every 1 × 10^4^ generations. Historical population expansion was inferred from COI + 16S rRNA haplotypes using a mismatch distribution analysis with 1000 bootstrap replications in Arlequin 3.1 [35]. Then, the sum of square deviations (SSD) was used to test the goodness of fit between the observed and the expected demographic models; Harpending’s Raggedness index (HRi) was applied to the statistical significance test [35]. 

To evaluate the extent of dispersal routes among populations in the hilly lineage of NSC, the coalescent based maximum likelihood method implemented in MIGRATE v. 3.6.4 was employed [38,39] using COI + 16S rRNA genes. The geographical distances between populations were measured using Google Earth v. 6.1 (1 October 2016). In addition, the COI gene was used to detect the influence of the ancestral haplotype on the dispersal inference of the hilly lineage of NSC at the LGM. The dataset analyzed included two combinations: (1) all populations and (2) populations excluding ancestral haplotypes, namely, major haplotype and the intermediate haplotypes connected to outgroups. In combination 2, population G was not included in the analysis because all the samples were included in the major haplotype. The 16S rRNA gene was not considered in this analysis because of its complicated ancestral haplotype structure. Population size Θ (Θ = *N_e_* μ, where *N_e_* is the effective population size and μ is the mutation rate) and migration rate *M* (*M* = *m*/μ; where *m* is the migration rate per generation) were estimated using *F*_ST_-calculation for start values and a starting tree by UPGMA for parameters. The migration rate (*M*) was estimated at a mutation rate of 2.69 × 10^−8^ per generation and 2.3 × 10^−8^ per generation for COI + 16S rRNA genes and the COI gene, respectively [31]. The parameters set for the analysis were 10 short chains with 100,000 recorded genealogies and 3 long chains with 50,000 recorded genealogies; both chains were sampled once every 100 steps, and the first 10,000 steps were discarded as burn-in.

## 3. Results

### 3.1. Sequence Composition of COI and 16S rRNA Genes

Both COI and 16S rRNA genes were successfully acquired for 171 specimens of *N*. *swinhoei*. The sequence lengths for COI and 16S rRNA genes were 686/669 bp and 550 bp, respectively. The average base compositions of G, A, T, and C were 16.5%, 30.8%, 30.9%, and 21.8% in the COI gene and 20.9%, 29.2%, 40.0%, and 9.9% in 16S rRNA gene, respectively. A total of 83 new sequences amplified in the present study have been deposited in GenBank under LC590052–LC590134 for the COI gene and LC590135–LC590217 for 16S rRNA gene.

### 3.2. Phylogenetic Analyses

**Figure 2:**
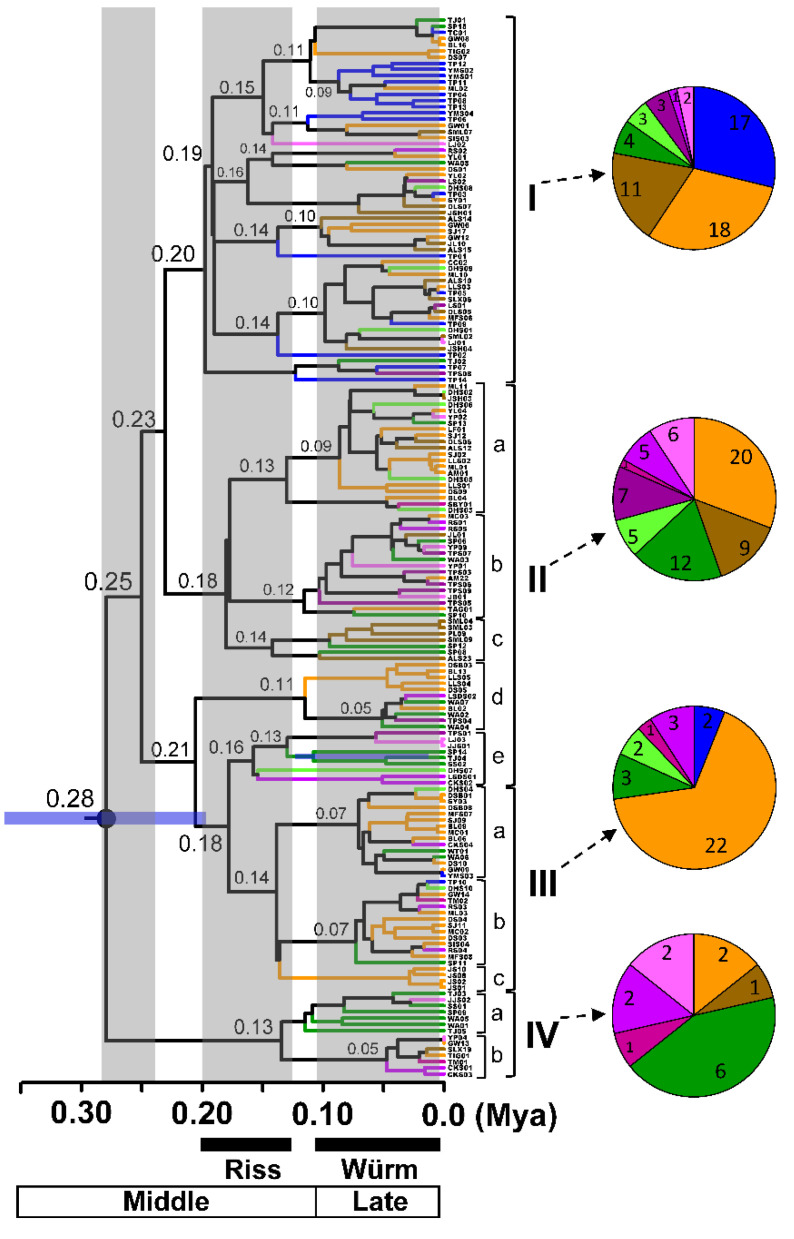
Calibration dating of the hilly lineage of NSC based on cytochrome oxidase subunit I (COI) + 16S rRNA genes. Populations denoted with different colors are the same as those in Figure 1. Pleistocene glaciation events are shaded in gray and labeled at the bottom [40].

Based on the origin time of approximately 0.2 Mya, phylogenetic inference using COI + 16S rRNA genes revealed four lineages in the hilly lineage of NSC, with several minor sublineages correlated with the geographical distribution of populations (Figure 2). Lineages II and III included populations across Taiwan Island, whereas population A was predominantly grouped in lineage I (17 of 19 individuals). Several sublineages were comprised of members from peculiar populations. For example, sublineages III-c and IV-a comprised of populations B and D; and sublineages II-c and II-b/IV-b comprised populations from southwestern and eastern Taiwan, respectively.

### 3.3. Divergence Time Calibration

Molecular dating based on COI + 16S rRNA genes (COI: 1.77%/lineage/Myr; 16S rRNA gene: 0.53%/lineage/Myr) showed that major lineage divergence occurred 0.18–0.28 Mya in the middle Pleistocene, and the subsequently formed sublineages diversified around the time of the LGM, which was 0.01–0.11 Mya (Figure 2). Phylogenetic inference based on COI revealed that the hilly lineage of NSC was originated about 0.15 Mya, all the population were genetically admixed as a major phylogroup without geographic association (Figure S1). A more divergent pattern was found in 16S rRNA gene (Figure S2), the hilly lineage originated approximately 0.67 Mya and the phylogenetic relationship was similar to the COI + 16S rRNA tree, which had four divergent lineages and a tendency of geographically genetic differentiation in both lineages and sublineages.

The substitution rates applied for insects, namely COI: 1.15%/lineage/Myr, 16S rRNA gene: 1%/lineage/Myr, revealed that the origin time of each lineage was approximately 0.3 Mya (Figure S3). Four major lineages were similar to Figure 2; both lineages and sublineages were found with geographic association. In the COI tree, the origin time of the hilly lineage of NSC could be dated to 0.22 Mya (Figure S4). While in the 16S rRNA gene based tree (Figure S5), the origin time was congruent with the COI + 16S rRNA tree, with the existence of four major lineages.

### 3.4. Haplotype Network and Possible Ancestral Haplotypes

**Figure 3:**
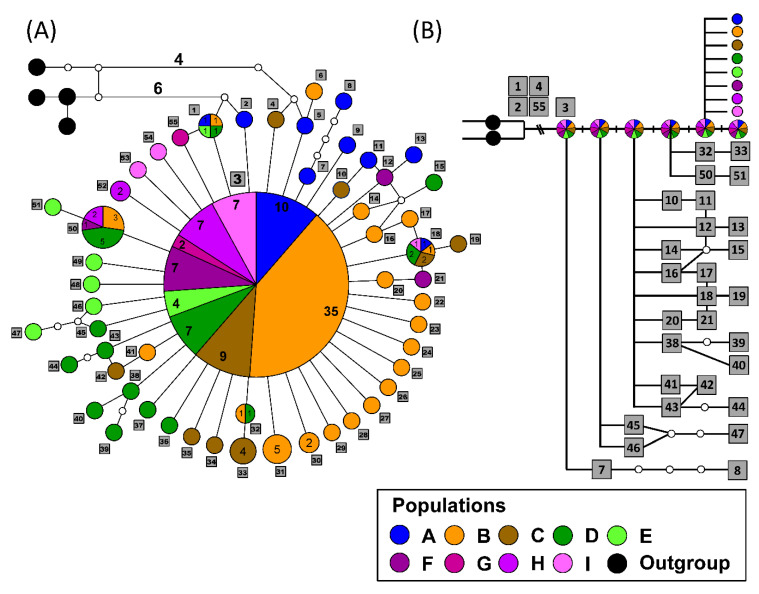
Haplotype network (**A**) and the possible evolution processes of ancestral and derived haplotypes according to the DELTRAN optimization (**B**) of the hilly lineage of NSC based on the COI gene. Populations are represented by different colors referring to the information given in Figure 1. Four individuals from the montane lineage are rooted. Each circle represents a haplotype, and the circle size is proportional to the number of individuals. The smallest circle corresponds to one individual, and the number of individuals of each population is marked on the shared haplotypes. The serial numbers of haplotypes were labeled in grey squares according to the population order from A to I.

**Figure 4:**
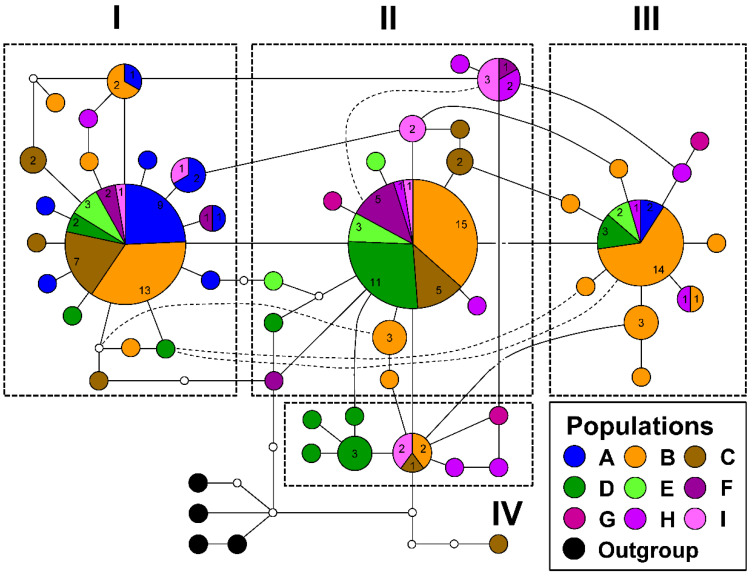
Haplotype network of the hilly lineage of NSC based on 16S rRNA gene. Haplogroups I to IV are grouped based on the phylogenetic inference. Populations are represented by different colors according to Figure 1. Four individuals from the montane lineage are rooted. Each circle represents a haplotype, and the circle size is proportional to the number of individuals. The smallest circle corresponds to one individual, and the number of individuals of each population is marked on the corresponding circle.

Haplotype network analyses of the hilly lineage of NSC revealed 55 and 50 haplotypes for COI and 16S rRNA genes, respectively. The COI network showed that the widespread *N*. *swinhoei* differs in eight substitution steps from the outgroups (Figure 3); and haplogroup IV in 16S rRNA gene was at least three substitution steps from the outgroups (Figure 4). In the COI gene, the network displayed a starlike shape centered around a major haplotype comprising 88 specimens from all populations. Of all the subsidiary haplotypes, a total of 50 mono-tip-extended haplotypes existed in each population, whereas four haplotypes were shared by at least two populations. 

In contrast to the COI gene, 16S rRNA gene was comprised four haplogroups, in consistence with the phylogenetic relationship and the timing of divergence at approximately 0.2 Mya (Figure 2 and Figure 4). Each of the major haplotypes in haplogroups I to III was shared by 5–7 populations. Although haplogroups I and II and haplogroups II and III were connected with only one substitution step, two to five substitution steps were found within each haplogroup. The present network structure revealed the following tendencies of geographical differentiation: (1) north‒south differentiation: 17/19 specimens from the northern population A were grouped in haplogroup I, and 12/25 and 5/10 individuals of the southern populations D and E, respectively, were found in haplogroup II; (2) 19 individuals from eastern Taiwan were found in haplogroup II, and haplogroup III is mainly composed of the northwestern population B; (3) haplogroup IV was composed of several geographically specific haplotypes from populations D, G, and H. 

The outgroup comparisons, phylogenetic relationships, and haplotype network relationships were used to infer the ancestral haplotypes of the hilly lineage in NSC. The earlier derived haplotypes were defined more ancestral than the later haplotypes. In the COI gene, the intermediate haplotypes between outgroups and the major haplotype revealed a possibility of being ancestral states. Many tip-extended haplotypes with specific geographical distributions derived from the major haplotype exhibited an ancestral feature of the major haplotype, which was that it had a widespread distribution. Based on the DELTRAN optimization, the simplified diagram in Figure 3B elucidated the possibility of arising order of ancestral haplotypes by comparing substitution steps with the outgroups. In 16S rRNA gene, the haplotype network was consistent with the phylogenetic relationship. Haplogroup IV had the closest distance to the outgroups, suggesting that the most ancestral haplotype might have existed approximately 0.28 Mya (Figure 2 and Figure 4). Similarly, the tip-extended haplotypes in 16S rRNA gene could be recognized as new mutations derived from the major haplotypes, though many intermediate haplotypes existed.

### 3.5. Nucleotide Diversity Indices

Nucleotide diversities (π) of all populations ranged from 0.0009 to 0.0029 for the COI gene and from 0.0019 to 0.0061 for 16S rRNA gene (Table 3). Nucleotide diversities of the COI gene were high in the western populations A, C, D, and E, and those from 16S rRNA gene were discovered in populations B, C, D, G, H, and I. Haplotype diversities (h, >0.5) were high in all populations in both COI and 16S rRNA genes. In neutrality tests, Tajima’s *D* analysis on the COI gene revealed significant negative values (*p* < 0.05) in populations A, B, C, D, F, and I, whereas significant negative values were found only in population A in 16S rRNA gene. Moreover, Fu’s *Fs* values from the COI gene revealed significant negative values (*p* < 0.05) in populations A, B, C, D, E, F, and I; in 16S rRNA gene, significant negative values were found in populations A, B, C, D, F, H, and I (Table 3). 

**Figure 5:**
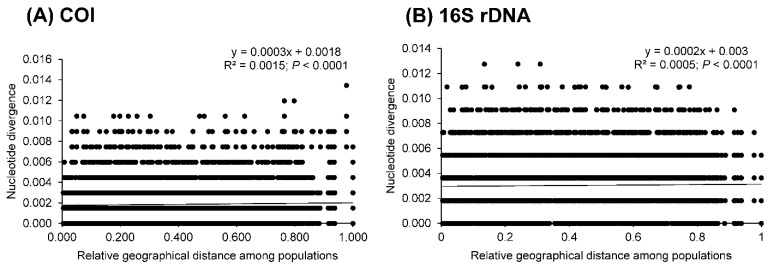
Relationships of nucleotide divergences to relative geographical distances between populations in the hilly lineage of NSC based on the COI gene (**A**) and 16S rRNA gene (**B**).

Although low genetic differentiation (*F*_ST_ < 0.05) was detected for most populations in the COI gene, the indices from 16S rRNA gene showed greater differentiation (Table 4). In 16S rRNA gene, population A showed moderate to high genetic differentiation from other populations with significance (*p* < 0.05), except population C. Populations from western Taiwan, namely populations B, C, D, and E, revealed moderate to high genetic differentiation from the eastern populations G and H. Moreover, slightly positive relationships between nucleotide divergence and relative geographical distances were observed in both genes (COI: *r* = 0.039, *p* < 0.0001; 16S rRNA gene: *r* = 0.022, *p* < 0.0001; Figure 5). In AMOVA, the components of variance within populations revealed high genetic admixtures, accounting for approximately 98% and 87% for COI and 16S rRNA genes, respectively (Table 5).

### 3.6. Population Demography

**Figure 6:**
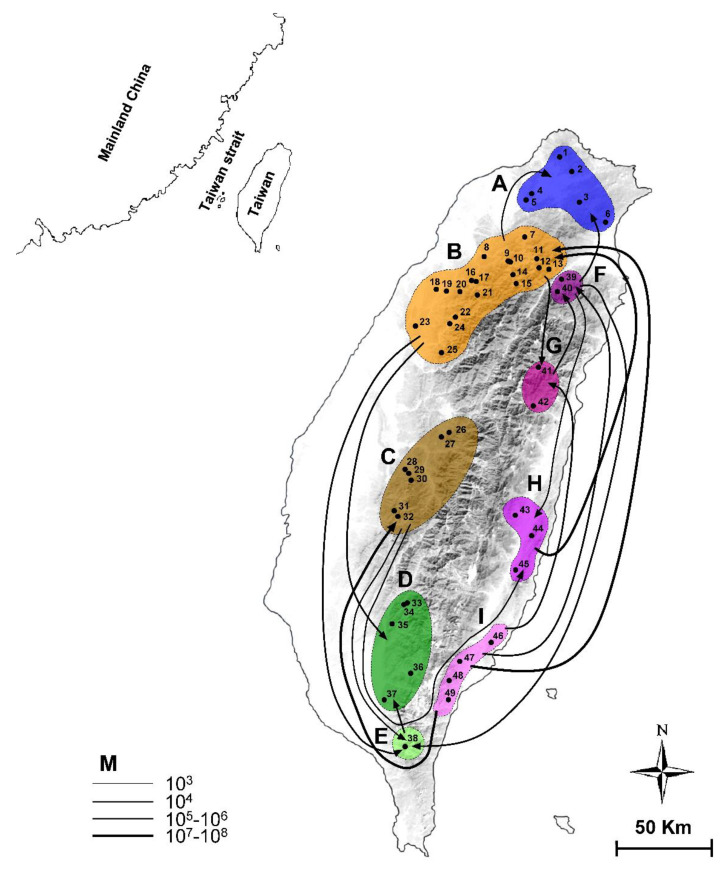
Dispersal inferences on the current demographic patterns for the hilly lineage of NSC by using COI + 16S rRNA genes. Nine populations were tentatively defined based on the topography of Taiwan Island. The migration rate (*M*) is shown by lines of different thicknesses at the bottom left.

The MIGRATE analysis revealed that the historic gene flow of widespread *N*. *swinhoei* occurred with some long-distance dispersal events (Figure 6 and Figure S6). Based on the analysis results for COI + 16S rRNA genes, the dispersal inference revealed that gene flow could be defined into three levels: I—gene flow between neighboring populations (25%); II—gene flow across one population (31.2%); and III—gene flow across two or more populations (43.8%) (Table 6, Figure 6). Both immigration and emigration frequently occurred among populations B, C, F, G, and H. Populations A and D only received immigrants from populations B/F and populations B/E, respectively. Population E mostly received gene flow from populations B, C, and F. However, for eastern population I, only emigration to western populations B and C and eastern populations F and G was detected.

In the COI gene, the dispersal levels among all populations were as follows: I—38.5%; II—38.5%; and III—23% (Table S3, Figure S6A). Both immigration and emigration were common in most populations. The northern populations A and F were observed to receive immigrants only from populations C/E and population H, respectively. In southern Taiwan, dispersal events frequently occurred between adjacent populations D/E and D/I. When the major and intermediate haplotypes to the outgroup were removed, the simulation results revealed that most dispersal occurred predominantly among adjacent populations, namely at dispersal levels I (50%), II (20%), and III (30%) (Table S3, Figure S6B). However, several dispersal events were detected from eastern to western Taiwan through both northern and southern routes.

**Figure 7:**
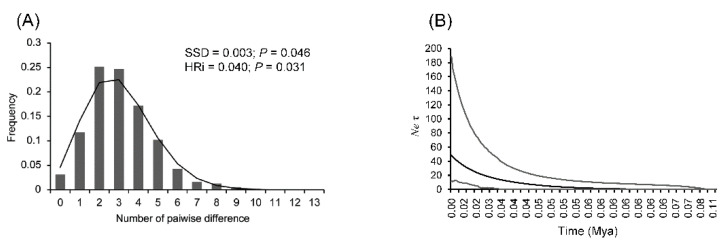
Mismatch distribution (**A**) and extended Bayesian skyline plots (**B**) of the hilly lineage of NSC inferred from COI + 16S rRNA genes. Statistical results are shown in the panel.

Geographical populations with unimodal mismatch distributions were perceived from COI + 16S rRNA genes (Figure 7A). The historical effective population size inferred from EBSP using COI + 16S rRNA genes revealed that the populations grew continuously since 0.06 Mya (Figure 7B).

## 4. Discussion

### 4.1. Implication of Genetic Variations on the Hilly Lineage of NSC

The inconsistent substitution patterns between COI and 16S rRNA gene are seldom addressed in extant organisms, though both genes are located in mitochondrial DNA. Generally, the faster evolving COI gene exhibits more genetic variations than the conserved 16S rRNA gene. However, our results reveal three major haplotypes in 16S rRNA gene and only one major haplotype in the COI gene (Figure 3 and Figure 4). Despite the more divergent pattern observed in 16S rRNA gene, the COI gene has more variable sites (52/669 bp) than 16S rRNA gene (25/550 bp). Moreover, slightly more haplotypes are found in the COI gene (55) than in 16S rRNA gene (50). Indeed, a study on bream fish indicated that hybridization events result in composition changes in mitochondrial genomes [41]. In the widespread *N*. *swinhoei*, frequent gene flow and introgression among populations and differentiated lineages accompanied with ancestral haplotype maintenance during the Riss and Würm glaciations might have caused the heredity pattern observed in this study [16,42].

Functional demands and substitution compensations also provide possible explanations for the inconsistent pattern between COI and 16S rRNA gene in *N*. *swinhoei*. The mitochondrial COI gene is a protein-coding gene that generally evolves under functional constraint with synonymous substitutions [40]. The secondary structure of the noncoding 16S rRNA gene not only maintains its translation function but also possibly accumulates compensation mutations [43]. Moreover, heteroplasmy in mitochondria might be an alternative hypothesis for the present genetic pattern in 16S rRNA gene [44,45].

Previous studies have pointed out that the low genetic variations in mitochondrial genes may be affected by the insufficient time for lineage sorting [46]. Recently, the increasing trend of using large amounts of loci obtained from genomic data, e.g., RAD-seq, has proposed the efficacy of delineating genetic differentiation among populations [47]. The application of genomic data may help us to document the detailed differentiation processes among populations for the hilly lineage of NSC in the future.

### 4.2. Ancestral Haplotype Determination

The network outgroup comparisons and frequency of major haplotypes provided helpful evidence to identify ancestral haplotypes in *N*. *swinhoei*. The sporadic haplotypes mediated between outgroups and major haplotypes in 16S rRNA and COI genes revealed their role in ancestral characteristics. These mediators and their closely related haplotypes might have been ancestral haplotypes, though less genetic drift occurred in the two genes of *N. swinhoei*. However, in studies on population genetics, major haplotypes have often been recognized as ancestral haplotypes for the derived tip-extended ones [48,49,50]. Owing to the weak flying capability and two-week adult lifespan of *N*. *swinhoei*, our results suggest that the tip-extended haplotypes have evolved recently in each local population. Therefore, the major haplotypes and intermediate ones closer to the outgroups were recognized as ancestral states that evolved prior the tip-extended haplotypes (Figure 3 and Figure 4). 

In the COI gene, the major haplotype was distributed across Taiwan with numerous tip-extended haplotypes in each local population, revealing the influence of Pleistocene glacial cycles. One possible scenario is that the ancestors of *N*. *swinhoei* with ancestral haplotype retention were widely distributed across Taiwan Island before the Riss glaciation and thereafter evolved in separate areas during the Riss–Würm glaciations, as reflected in the divergent network of 16S rRNA gene (Figure 2 and Figure 3). Stable population growth of the major haplotype and the new mutations, namely tip-extended haplotypes, simultaneously occurred in each population approximately 0.06 Mya during the LGM (Figure 7B). The major and geographically derived haplotypes were therefore found to coexist in each population. Otherwise, the extant *N*. *swinhoei* could not have accumulated many local mutations after the recent population expansion during the LGM.

### 4.3. Evolutionary History of Widespread N. swinhoei

The differentiation processes of the hilly lineage of NSC are the first phylogeographical study to investigate the influence of 100,000-year Pleistocene glacial cycles on the subtropical Taiwan Island [2,3,8]. Our data indicate that the hilly lineage originated prior to 0.28 Mya [16], and *N. swinhoei* may have been isolated in several refugia following glaciation 0.18–0.28 Mya, as suggested by the divergent lineages (Figure 2). Afterwards, several geographically associated sublineages suggest effects of refugia isolation approximately 0.10–0.20 Mya during the Riss glaciation. One possible evolutionary explanation is repeated isolation in and expansion from refugia during the periodical Riss–Würm glaciations, which would result in the coexistence of ancestral retentions and new mutations in the *N*. *swinhoei* populations (Figure 6).

The phylogenetic relationships and genetic composition data offer useful information to deduce the possible refugia for the widespread *N*. *swinhoei*. Based on our phylogenetic hypothesis (Figure 2), several geographically relevant lineages and sublineages, namely, I, II-c, III-c, and IV-a, may indicate possible refugia during Pleistocene glaciations. For lineage I, a possible refugium may have occurred around the Taipei basin in northern Taiwan, as supported by the following evidence: (1) 17 of 19 specimens from population A were grouped in this lineage; (2) the *F*_ST_ values of 16S rRNA gene indicated moderate to high genetic differentiation among population A and the other populations (Table 4); and (3) population A received immigrants only from adjacent populations B and F (Figure 6). The possible refugium was deduced to be located near the Datun hills, where hot springs are usually found [51]. For population B, the presence of several specific groups in each sublineage (II-d and III-c), high genetic diversity, and nucleotide diversity suggest that several possible refugia existed in northwestern Taiwan (Table 3). Studies have proposed that the mountain area around the Syueshan Mountain Range (SMR) is a possible refugium for the beech *Castanopsis carlesii*, oak *Quercus glauca*, and NSC [16,52,53]. A possible refugium might be situated near the low-altitude area of SMR, namely, the geographical region of population B. Because sublineage IV-a was mainly composed of population D (Figure 2), one possible refugium might be located in southwestern or southern Taiwan, which has been proposed as the refugium for beech *Castanopsis carlesii*, oak *Quercus glauca*, and *Michelia formosana* [52,53,54]. Southern Taiwan, due to its tropical and warm climate, can provide a suitable living environment for southern *N*. *swinhoei* during glacial cycles. Currently, a similar overwinter ecological feature is observed in nymphalid *Euploea* butterflies [55,56]. 

Finally, the detection of many specific haplotypes in eastern populations suggests the profound effects of CMR hindrance and the existence of putative refugia in eastern Taiwan, which has been demonstrated for frogs, toads, and crickets [15,21,22]. The existence of divergent haplotypes in eastern populations also suggests that their ancestral origin occurred before the Riss glaciation and was then isolated by the CMR. During the LGM, the stable population growth of *N*. *swinhoei* might have promoted frequent gene flow between adjacent populations, namely, between southern populations D and I and between northern populations A and F (Figure S6).

### 4.4. Historical Dispersal of the Widespread N. swinhoei

The altitudinal distribution of *N*. *swinhoei* ranges from 200 to 1500 m and the specific genetic structure in eastern Taiwan suggest the effects of CMR hindrance on dispersal routes [16]. The 16S rRNA haplotype network confirms the historical dispersal routes for western *N*. *swinhoei* to eastern Taiwan through both northern and southern low-altitude mountains before Riss glaciation, as suggested by the close relationships of several haplotypes between eastern and western populations (Figure 2 and Figure 4); thereafter, these stag beetles were isolated in the eastern refugia. The southern dispersal route has been demonstrated in several organisms, including toad (*Bufo bankorensis*), crabs (*Candidiopotamon rathbunae*/*Geothelphusa*), common cricket (*Loxoblemmus appendicularis*), damselfly (*Euphaea formosa*), swallowtail butterfly (*Troides aeacus formosanus*), and the southeastern lineage of NSC [15,16,17,21,57,58,59]. The northern dispersal route was deduced for the damselfly *Euphaea formosa*, on the basis of genetic structure analysis [59]. During the LGM, the eastern *N*. *swinhoei* may have had stable growth and dispersed back to the western region through lower mountains in northern and southern Taiwan (Figure 7 and Figure S6). 

Owing to the weak dispersal capability and two-week adult lifespan of NSC, the long-distance dispersal events from the eastern populations to western Taiwan might have been induced by the retention of the ancestral haplotypes in both 16S rRNA and COI genes; the intermediated haplotypes in 16S rRNA gene with geographical association could also account for the long-distance dispersal events (Figure 6 and Figure S6). For instances, several long-distance dispersals were not recovered if the major haplotype of the COI gene was excluded from the analysis (Figure S6). Thus, the shared haplotypes of H1, H18, H32, and H50 are also believed relevant to the long-distance dispersal events (Figure S6B). By far, of the nearly 100 phylogeographical studies in Taiwan, this is the first to reveal westward dispersal from ancestral lineages isolated in eastern Taiwan.

## 5. Conclusions

The present study revealed the peculiar genetic structure of the hilly lineage of NSC and investigated possible phylogeographical scenarios on the subtropical Taiwan Island. The inconsistent evolutionary patterns between COI and 16S rRNA gene were likely the result of functional constraint, biological features of 2-week adult life span and weak flying capability, and secondary contact after stable demographic growth during the LGM. Together, these factors resulted in ancestral haplotype maintenance and new mutations originating in different genealogical periods and geographical populations. Four possible refugia were identified in northern, northwestern, southern, and eastern Taiwan. The southern and northern dispersal routes to eastern Taiwan are congruent with those identified in previous studies, although the extent of refugia and dispersal duration may reveal a different differentiation history. The westward dispersion from eastern populations due to population expansion during the LGM is the first reported case in the phylogeographical studies in Taiwan.

## Figures and Tables

**Table 1:** Collection localities of the hilly lineage of *Neolucanus swinhoei* complex across Taiwan Island.

Pop	Collection Locality											
A	1	Beitou	2	Neihu	3	Shihding	4	Shulin	5	Yingge	6	Toucheng
B	7	Luofu	8	Dashanbei	9	Daosia	10	Yulao	11	Lalashan	12	Sihleng
	13	Mingchih	14	Taigang	15	Jianshih	16	Bailan	17	Cingcyuan	18	Mingfongshan
	19	Sianshan	20	Luchang	21	Guanwu	22	Syuejian	23	Sanyi	24	Tiangou
	25	Anma Mountain										
C	26	Puli	27	Sun Moon Lake	28	Jhulin Village	29	Dalunshan	30	Shanlinsi	31	Jhangshuhu
	32	Alishan										
D	33	Shihshan	34	Tengjhih	35	Shanping	36	Wutai	37	Wanan		
E	38	Dahanshan										
F	39	Leshuei	40	Taipingshan								
G	41	Sinbaiyang	42	Tongmen								
H	43	Rueisuei	44	Chihkeshan	45	Lioushihdanshan						
I	46	Yanping	47	Lijia	48	Jhihben	49	Jinjhenshan				

**Table 2:** Primers and amplification sizes for each gene in this study.

Gene	Primer	Sequence 5’→ 3’	Size (bp)	References
COI	CI-46Coleoptera (+)	AACCATAAAGATATTGGAAC	686	Tsai et al. [16]
	CI-Neo-F (+)	AACATTATACTTCCTRCTAGGTA	669	
	CI-731Coleoptera (−)	CCAAAAAATCAAAATAAATGTTG		
16S rRNA	16SR21 (+)	GCCTGTTTATCAAAAACAT	550	Yeh et al. [29]
	16S22 (−)	CCGGTCTGAACTCAGATCA		

**Table 3:** Genetic diversity and neutrality test of COI and 16S rRNA genes in populations of the hilly lineage of NSC.

Pop	N	S		*π*		Hn		*h*		*T_D_*		*Fs*	
		COI	16S	COI	16S	COI	16S	COI	16S	COI	16S	COI	16S
A	19	13	9	0.0023	0.0022	10	9	0.7368	0.7778	**−2.13875**	**−1.74253**	**−6.19070**	**−5.81418**
B	62	20	11	0.0014	0.0030	21	17	0.6779	0.8509	**−2.42149**	−0.76986	**−26.82927**	**−10.85315**
C	21	10	12	0.0023	0.0035	9	9	0.7952	0.8429	**−1.50025**	−1.42923	**−4.21905**	**−3.36040**
D	25	17	9	0.0028	0.0030	14	10	0.8933	0.7933	**−2.05377**	−0.78729	**−10.17280**	**−4.62452**
E	10	8	5	0.0029	0.0026	7	5	0.8667	0.8444	−1.39868	−0.78318	**−3.42350**	−1.39262
F	10	4	4	0.0012	0.0019	4	5	0.5333	0.7556	**−1.66706**	−0.65748	**−1.34464**	**−2.09575**
G	3	1	5	0.0010	0.0061	2	3	0.6667	1.0000	0.00000	0.00000	0.20067	−0.07696
H	11	2	8	0.0010	0.0046	3	10	0.5818	0.9818	−0.12670	−0.72380	−0.21110	**−7.74072**
I	10	3	4	0.0009	0.0030	4	6	0.5333	0.8889	**−1.56222**	0.39804	**−1.96374**	**−2.36445**

**N:** number of individuals; **S:** number of segregating sites; ***π*:** nucleotide diversity; **Hn:** haplotype number; ***h*:** haplotype diversity; ***T_D_*:** Tajima’s *D* test; ***Fs*****:** Fu’s *Fs* test. Significant values with *p* < 0.05 are in bold.

**Table 4:** Population pairwise *F*_ST_ for COI (bottom left) and 16S rRNA gene (top right) for the hilly lineage of NSC.

	16S	A	B	C	D	E	F	G	H	I
COI										
A		-	**0.2172**	**0.0805**	**0.2878**	**0.1940**	**0.2098**	**0.4767**	**0.3572**	**0.2841**
B		**0.0231**	-	**0.0992**	**0.0622**	−0.0019	**0.0631**	0.2072	**0.1441**	**0.1217**
C		0.0387	**0.0637**	-	**0.0978**	0.0262	0.0036	**0.2743**	**0.2022**	**0.0899**
D		**0.0215**	**0.0319**	**0.0408**	-	0.0222	0.0427	0.2068	**0.1497**	0.0736
E		0.0314	**0.0729**	**0.0694**	0.0200	-	−0.0200	**0.2312**	**0.1315**	0.0873
F		−0.0304	−0.0180	0.0162	−0.0309	0.0177	-	**0.2729**	**0.1321**	0.0454
G		−0.1029	−0.0430	−0.0447	−0.0914	−0.0958	−0.0224	-	−0.1125	0.0899
H		0.0239	0.0192	**0.0645**	−0.0174	0.0388	0.0010	0.0547	-	0.0537
I		−0.0184	−0.0133	0.0129	−0.0040	0.0342	−0.0294	0.0137	0.0543	-

Significant values with *p* < 0.05 are in bold.

**Table 5:** Analysis of molecular variance (AMOVA) of COI and 16S rRNA genes in populations of the hilly lineage of NSC.

Gene	Source of Variation	Df	Sum of Squares	Variance Components	Variation (%)
COI	Among populations	8	6.923	0.01464	2.33
	Within populations	162	99.370	0.61339	97.67
16S rRNA	Among populations	8	23.398	0.12179	12.81
	Within populations	162	134.304	0.82904	87.19

**Table 6:** Frequency of dispersal inference estimated through COI + 16S rRNA genes.

Level	No. of Migration	Frequency (%)
I	4	25
II	5	31.2
III	7	43.8

## Data Availability

The sequences amplified in this study have been deposited in GenBank. Accession numbers and relevant information of voucher specimens are listed in Table S1.

## Supplementary Materials

The following are available online at https://www.mdpi.com/2075-4450/12/3/227/s1, Figure S1: Calibration dating of the hilly lineage of NSC based on the COI gene using 1.77%/lineage per million years (Myr). Populations denoted with different colors are the same as those in Figure 1. Pleistocene glaciation events are shaded in gray and labeled at the bottom [40]. Figure S2: Calibration dating of the hilly lineage of NSC based on 16S rRNA gene using 0.53%/lineage/Myr. Populations denoted with different colors are the same as those in Figure 1. Pleistocene glaciation events are shaded in gray and labeled at the bottom [40]. Figure S3: Calibration dating of the hilly lineage of NSC based on COI + 16S rRNA genes (COI: 1.15%/lineage/Myr; 16S rRNA gene: 1%/lineage/Myr). Populations denoted with different colors are the same as those in Figure 1. Pleistocene glaciation events are shaded in gray and labeled at the bottom [40]. Figure S4: Calibration dating of the hilly lineage of NSC based on the COI gene using 1.15%/lineage per million years (Myr). Populations denoted with different colors are the same as those in Figure 1. Pleistocene glaciation events are shaded in gray and labeled at the bottom [40]. Figure S5: Calibration dating of the hilly lineage of NSC based on 16S rRNA gene using 1%/lineage/Myr. Populations denoted with different colors are the same as those in Figure 1. Pleistocene glaciation events are shaded in gray and labeled at the bottom [40]. Figure S6: Dispersal inferences on the current demographic patterns for the hilly lineage of *Neolucanus* *swinhoei* complex (A) and the exclusion of ancestral haplotypes (B) by using the COI gene. The migration rate (*M*) is denoted by lines of different thicknesses at the bottom left. Haplotype compositions including major, unique, and some shared haplotypes for each population are shown in panel A. Table S1: Taxon ID, collection locality, Global Positioning System (GPS) coordinates, and accession numbers of COI and 16S rRNA genes for each *Neolucanus* stag beetle. Table S2: Immigration rates of widespread *N*. *swinhoei* among nine populations throughout Taiwan Island estimated using MIGRATE. Table S3: Frequency of dispersal inference estimated using the COI gene.
