# Supplementary material for: Ancestral Haplotype Retention and Population Expansion Determine the Complicated Population Genetic Structure of the Hilly Lineage of Neolucanus swinhoei Complex (Coleoptera, Lucanidae) on the Subtropical Taiwan Island
Source: Insects. 2021 Mar 5;12(3):227. doi: 10.3390/insects12030227 (PMC7999642; doi:10.3390/insects12030227)
Supplement: Supplementary file 1 [file insects-12-00227-s001.zip › Table S2.docx]

Table S2. Immigration rates of widespread *N*. *swinhoei* among nine populations throughout Taiwan Island estimated using MIGRATE

| **Population** | **Θ** | **1, +** | **2, +** | **3, +** | **4, +** | **5, +** | **6, +** | **7, +** | **8, +** | **9, +** |
| --- | --- | --- | --- | --- | --- | --- | --- | --- | --- | --- |
| **1: A** | 0.0027 | - | 20200 | 0 | 0 | 0 | 130000 | 0 | 0 | 0 |
| **2: B** | 0.0002 | 0 | - | 0 | 0 | 0 | 0 | 0 | 7.96E+07 | 1.84E+07 |
| **3: C** | 0.0002 | 0 | 0 | - | 0 | 0 | 0 | 0 | 0 | 2.26E+07 |
| **4: D** | 0.0019 | 0 | 170000 | 0 | - | 880000 | 0 | 0 | 0 | 0 |
| **5: E** | 0.0236 | 0 | 238000 | 38200 | 0 | - | 204000 | 0 | 0 | 0 |
| **6: F** | 0.0001 | 0 | 0 | 0 | 0 | 0 | - | 1.54E+06 | 0 | 7.19E+06 |
| **7: G** | 0.0001 | 0 | 859000 | 0 | 0 | 0 | 0 | - | 0 | 113000 |
| **8: H** | 0.0102 | 0 | 0 | 31700 | 0 | 0 | 71700 | 0 | - | 0 |
| **9: I** | 0.0120 | 0 | 0 | 0 | 0 | 0 | 0 | 0 | 0 | - |

**Θ:** population size; **“+”**: receiving population; pops A to I are the same in Fig. 1.
