# Supplementary material for: Ancestral Haplotype Retention and Population Expansion Determine the Complicated Population Genetic Structure of the Hilly Lineage of Neolucanus swinhoei Complex (Coleoptera, Lucanidae) on the Subtropical Taiwan Island
Source: Insects. 2021 Mar 5;12(3):227. doi: 10.3390/insects12030227 (PMC7999642; doi:10.3390/insects12030227)
Supplement: Supplementary file 1 [file insects-12-00227-s001.zip › Table S3.docx]

|  | **All populations** | | **Remove ancestral haplotypes** | |
| --- | --- | --- | --- | --- |
| **Level** | **No. of migration** | **Frequency (%)** | **No. of migration** | **Frequency (%)** |
| **I** | 5 | 38.5 | 5 | 50 |
| **II** | 5 | 38.5 | 2 | 20 |
| **III** | 3 | 23.0 | 3 | 30 |

Table S3. The frequency of dispersal inference estimated through the COI gene

I: gene flow between neighboring populations; II: across over one population; III: across two or more populations.
