# Supplementary material for: Ancestral Haplotype Retention and Population Expansion Determine the Complicated Population Genetic Structure of the Hilly Lineage of Neolucanus swinhoei Complex (Coleoptera, Lucanidae) on the Subtropical Taiwan Island
Source: Insects. 2021 Mar 5;12(3):227. doi: 10.3390/insects12030227 (PMC7999642; doi:10.3390/insects12030227)
Supplement: Supplementary file 1 [file insects-12-00227-s001.zip › Table S1.docx]

Table S1. Taxon ID, collection locality, Global Positioning System (GPS) coordinates, and accession numbers of COI and 16S rRNA gene for each *Neolucanus* stag beetle

| **Pop** | **Taxon** | **Voucher No.** | **ID** | **Collecting Locality** | **GPS-latitude** | **GPS-longitude** | **COI** | **16S rRNA** |
| --- | --- | --- | --- | --- | --- | --- | --- | --- |
| **A** | *N*. *swinhoei* | Luc103 | TP01 | Neihu, Taipei City | 25.098746° | 121.599438° | AB762221 | AB762601 |
|  | *N*. *swinhoei* | Luc106 | TP02 | Yingge, New Taipei City | 24.985206° | 121.353294° | AB762222 | AB762602 |
|  | *N*. *swinhoei* | Luc130 | TP03 | Beitou, Taipei City | 25.181690° | 121.532368° | AB762223 | AB762603 |
|  | *N*. *swinhoei* | Luc621 | TP04 | Shihding, New Taipei City | 24.683833° | 121.620417° | AB762255 | AB762635 |
|  | *N*. *swinhoei* | Luc622 | TP05 | Shihding, New Taipei City | 24.683833° | 121.620417° | AB762256 | AB762636 |
|  | *N*. *swinhoei* | Luc623 | TP06 | Shihding, New Taipei City | 24.683833° | 121.620417° | AB762257 | AB762637 |
|  | *N*. *swinhoei* | Luc624 | TP07 | Shihding, New Taipei City | 24.683833° | 121.620417° | AB762258 | AB762638 |
|  | *N*. *swinhoei* | Luc625 | TP08 | Shihding, New Taipei City | 24.683833° | 121.620417° | AB762259 | AB762639 |
|  | *N*. *swinhoei* | Luc685 | TP09 | Shulin, New Taipei City | 25.050080° | 121.403447° | AB762260 | AB762640 |
|  | *N*. *swinhoei* | Luc686 | TP10 | Shulin, New Taipei City | 25.050080° | 121.403447° | AB762261 | AB762641 |
|  | *N*. *swinhoei* | Luc687 | TP11 | Shulin, New Taipei City | 25.050080° | 121.403447° | AB762262 | AB762642 |
|  | *N*. *swinhoei* | Luc688 | TP12 | Shulin, New Taipei City | 25.050080° | 121.403447° | AB762263 | AB762643 |
|  | *N*. *swinhoei* | Luc689 | TP13 | Shulin, New Taipei City | 25.050080° | 121.403447° | AB762264 | AB762644 |
|  | *N*. *swinhoei* | Luc690 | TP14 | Shulin, New Taipei City | 25.050080° | 121.403447° | AB762265 | AB762645 |
|  | *N*. *swinhoei* | Luc1252 | YMS01 | Yangmingshan, Taipei City | 25.181690° | 121.532368° | LC590052 | LC590135 |
|  | *N*. *swinhoei* | Luc1253 | YMS02 | Yangmingshan, Taipei City | 25.181690° | 121.532368° | LC590053 | LC590136 |
|  | *N*. *swinhoei* | Luc1254 | YMS03 | Yangmingshan, Taipei City | 25.181690° | 121.532368° | LC590054 | LC590137 |
|  | *N*. *swinhoei* | Luc1256 | YMS04 | Yangmingshan, Taipei City | 25.181690° | 121.532368° | LC590055 | LC590138 |
|  | *N*. *swinhoei* | Luc1039 | TC01 | Toucheng, Provincial highway No. 9, Yilan | 24.868758° | 121.772013° | LC074637 | LC075138 |
| **B** | *N*. *doro doro* | Luc183 | AM01 | Mt. Anma, Taichung City | 24.227823° | 120.970330° | AB762102 | AB762476 |
|  | *N*. *swinhoei* | Luc446 | AM22 | Mt. Anma, Taichung City | 24.241802° | 120.943710° | AB762239 | AB762619 |
|  | *N*. *doro horaguchii* | Luc312 | SJ02 | Syuejian, Miaoli County | 24.409468° | 121.005077° | AB762157 | AB762531 |
|  | *N*. *doro horaguchii* | Luc357 | SJ09 | Syuejian, Miaoli County | 24.409468° | 121.005077° | AB762164 | AB762538 |
|  | *N*. *doro horaguchii* | Luc363 | SJ11 | Syuejian, Miaoli County | 24.409468° | 121.005077° | AB762166 | AB762540 |
|  | *N*. *doro horaguchii* | Luc366 | SJ12 | Syuejian, Miaoli County | 24.409468° | 121.005077° | AB762167 | AB762541 |
|  | *N*. *doro horaguchii* | Luc371 | SJ17 | Syuejian, Miaoli County | 24.409468° | 121.005077° | AB762172 | AB762546 |
|  | *N*. *doro horaguchii* | Luc416 | GW01 | Guanwu, Hsinchu County | 24.525874° | 121.114176° | AB762124 | AB762498 |
|  | *N*. *doro horaguchii* | Luc433 | GW06 | Guanwu, Hsinchu County | 24.525874° | 121.114176° | AB762129 | AB762503 |
|  | *N*. *doro horaguchii* | Luc435 | GW08 | Guanwu, Hsinchu County | 24.525874° | 121.114176° | AB762131 | AB762505 |
|  | *N*. *doro horaguchii* | Luc436 | GW09 | Guanwu, Hsinchu County | 24.525874° | 121.114176° | AB762132 | AB762506 |
|  | *N*. *doro horaguchii* | Luc439 | GW12 | Guanwu, Hsinchu County | 24.525874° | 121.114176° | AB762135 | AB762509 |
|  | *N*. *doro horaguchii* | LucAF021 | GW13 | Guanwu, Hsinchu County | 24.525874° | 121.114176° | AB762136 | AB762510 |
|  | *N*. *doro horaguchii* | LucAF023 | GW14 | Guanwu, Hsinchu County | 24.525874° | 121.114176° | AB762137 | AB762511 |
|  | *N*. *doro horaguchii* | Luc491 | JS01 | Jianshih, Hsinchu County | 24.572169° | 121.308506° | AB762153 | AB762527 |
|  | *N*. *doro horaguchii* | Luc492 | JS02 | Jianshih, Hsinchu County | 24.572169° | 121.308506° | AB762154 | AB762528 |
|  | *N*. *doro horaguchii* | Luc1107 | JS08 | Jianshih, Hsinchu County | 24.572169° | 121.308506° | LC590056 | LC590139 |
|  | *N*. *doro horaguchii* | Luc1109 | JS10 | Jianshih, Hsinchu County | 24.572169° | 121.308506° | LC590057 | LC590140 |
|  | *N*. *swinhoei* | Luc404 | ML01 | Luchang, Miaoli County | 24.540834° | 121.027512° | AB762231 | AB762611 |
|  | *N*. *swinhoei* | Luc405 | ML02 | Luchang, Miaoli County | 24.540834° | 121.027512° | AB762232 | AB762612 |
|  | *N*. *swinhoei* | Luc406 | ML03 | Luchang, Miaoli County | 24.540834° | 121.027512° | AB762233 | AB762613 |
|  | *N*. *swinhoei* | Luc590 | ML10 | Sanyi, Miaoli County | 24.374512° | 120.798065° | AB762247 | AB762627 |
|  | *N*. *swinhoei* | Luc595 | ML11 | Mingchih, Yilan County | 24.649402 | 121.423068 | AB762248 | AB762628 |
|  | *N*. *swinhoei* | Luc804 | LLS01 | Tamanshan, Taoyuan City | 24.698373° | 121.422129° | LC074629 | LC075130 |

Table S1 (continued)

| **Pop** | **Taxon** | **Voucher No.** | **ID** | **Collecting Locality** | **GPS-latitude** | **GPS-longitude** | **COI** | **16S rRNA** |
| --- | --- | --- | --- | --- | --- | --- | --- | --- |
| **B** | *N*. *swinhoei* | Luc808 | LLS02 | Tamanshan, Taoyuan City | 24.698373° | 121.422129° | LC074630 | LC075131 |
|  | *N*. *swinhoei* | Luc809 | LLS03 | Tamanshan, Taoyuan City | 24.698373° | 121.422129° | LC074631 | LC075132 |
|  | *N*. *swinhoei* | Luc812 | LLS04 | Lalashan, Taoyuan City | 24.698373° | 121.422129° | LC590058 | LC590141 |
|  | *N*. *swinhoei* | Luc814 | LLS05 | Lalashan, Taoyuan City | 24.698373° | 121.422129° | LC590059 | LC590142 |
|  | *N*. *swinhoei* | Luc721 | LF01 | Luofu, Taoyuan City | 24.797780° | 121.360944° | LC590060 | LC590143 |
|  | *N*. *swinhoei* | Luc949 | DS01 | Daosia, Hsinchu County | 24.669913° | 121.264780° | LC590061 | LC590144 |
|  | *N*. *swinhoei* | Luc951 | DS03 | Daosia, Hsinchu County | 24.669913° | 121.264780° | LC074632 | LC075133 |
|  | *N*. *swinhoei* | Luc952 | DS04 | Daosia, Hsinchu County | 24.669913° | 121.264780° | LC074633 | LC075134 |
|  | *N*. *swinhoei* | Luc953 | DS05 | Daosia, Hsinchu County | 24.669913° | 121.264780° | LC590062 | LC590145 |
|  | *N*. *swinhoei* | Luc978 | DS07 | Daosia, Hsinchu County | 24.669913° | 121.264780° | LC590063 | LC590146 |
|  | *N*. *swinhoei* | Luc995 | DS09 | Daosia, Hsinchu County | 24.669913° | 121.264780° | LC590064 | LC590147 |
|  | *N*. *swinhoei* | Luc996 | DS10 | Daosia, Hsinchu County | 24.669913° | 121.264780° | LC590065 | LC590148 |
|  | *N*. *swinhoei* | Luc920 | YL01 | Yulao, Hsinchu County | 24.664208° | 121.277522° | LC590066 | LC590149 |
|  | *N*. *swinhoei* | Luc921 | YL02 | Yulao, Hsinchu County | 24.664208° | 121.277522° | LC590067 | LC590150 |
|  | *N*. *swinhoei* | Luc957 | YL04 | Yulao, Hsinchu County | 24.664208° | 121.277522° | LC074634 | LC075135 |
|  | *N*. *swinhoei* | Luc963 | MC01 | Mingchih, Yilan County | 24.648867° | 121.466209° | LC074635 | LC075136 |
|  | *N*. *swinhoei* | Luc964 | MC02 | Mingchih, Yilan County | 24.648867° | 121.466209° | LC074636 | LC075137 |
|  | *N*. *swinhoei* | Luc965 | MC03 | Mingchih, Yilan County | 24.648867° | 121.466209° | LC590068 | LC590151 |
|  | *N*. *swinhoei* | Luc899 | BL02 | Bailan, Hsinchu County | 24.582942° | 121.082982° | LC590069 | LC590152 |
|  | *N*. *swinhoei* | Luc914 | BL04 | Bailan, Hsinchu County | 24.582942° | 121.082982° | LC590070 | LC590153 |
|  | *N*. *swinhoei* | Luc994 | BL06 | Bailan, Hsinchu County | 24.582942° | 121.082982° | LC590071 | LC590154 |
|  | *N*. *swinhoei* | Luc900 | BL09 | Bailan, Hsinchu County | 24.582942° | 121.082982° | LC590072 | LC590155 |
|  | *N*. *swinhoei* | Luc904 | BL13 | Bailan, Hsinchu County | 24.582942° | 121.082982° | LC590073 | LC590156 |
|  | *N*. *swinhoei* | Luc909 | BL16 | Bailan, Hsinchu County | 24.582942° | 121.082982° | LC590074 | LC590157 |
|  | *N*. *swinhoei* | Luc976 | TAG01 | Taigang, Hsinchu County | 24.613537° | 121.296957° | LC590075 | LC590158 |
|  | *N*. *swinhoei* | Luc985 | TIG01 | Tiangou, Hsinchu County | 24.389748° | 120.972579° | LC590076 | LC590159 |
|  | *N*. *swinhoei* | Luc1158 | TIG02 | Tiangou, Hsinchu County | 24.389748° | 120.972579° | LC590077 | LC590160 |
|  | *N*. *swinhoei* | Luc990 | CC02 | Cingcyuan, Hsinchu County | 24.573451° | 121.102287° | LC590078 | LC590161 |
|  | *N*. *swinhoei* | Luc1005 | DSB01 | Dashanbei, Hsinchu County | 24.695564° | 121.145151° | LC590079 | LC590162 |
|  | *N*. *swinhoei* | Luc1007 | DSB03 | Dashanbei, Hsinchu County | 24.695564° | 121.145151° | LC590080 | LC590163 |
|  | *N*. *swinhoei* | Luc1012 | DSB08 | Gaofong, Hsinchu County | 24.695564° | 121.145151° | LC590081 | LC590164 |
|  | *N*. *swinhoei* | Luc1036 | SY01 | Sanyi, Miaoli County | 24.374512° | 120.798065° | LC590082 | LC590165 |
|  | *N*. *swinhoei* | Luc1228 | SY03 | Sanyi, Miaoli County | 24.374512° | 120.798065° | LC590083 | LC590166 |
|  | *N*. *swinhoei* | Luc1225 | SIS03 | Sianshan, Miaoli County | 24.535578° | 120.952975° | LC590084 | LC590167 |
|  | *N*. *swinhoei* | Luc1226 | SIS04 | Sianshan, Miaoli County | 24.535578° | 120.952975° | LC590085 | LC590168 |
|  | *N*. *swinhoei* | Luc1243 | MFS06 | Mingfongshan, Miaoli County | 24.543125° | 120.898529° | LC590086 | LC590169 |
|  | *N*. *swinhoei* | Luc1244 | MFS07 | Mingfongshan, Miaoli County | 24.543125° | 120.898529° | LC590087 | LC590170 |
|  | *N*. *swinhoei* | Luc1245 | MFS08 | Mingfongshan, Miaoli County | 24.543125° | 120.898529° | LC590088 | LC590171 |
| **C** | *N*. *doro doro* | Luc251 | SLX06 | Shanlinsi, Nantou County | 23.656069° | 120.776446° | AB762179 | AB762553 |
|  | *N*. *doro doro* | Luc293 | SLX19 | Shanlinsi, Nantou County | 23.656069° | 120.776446° | AB762180 | AB762554 |
|  | *N*. *doro doro* | Luc424 | SML02 | Sun Moon Lake, Nantou County | 23.845109° | 120.924715° | AB762196 | AB762570 |
|  | *N*. *doro doro* | Luc425 | SML03 | Sun Moon Lake, Nantou County | 23.845109° | 120.924715° | AB762197 | AB762571 |
|  | *N*. *doro doro* | Luc466 | SML04 | Sun Moon Lake, Nantou County | 23.845109° | 120.924715° | AB762198 | AB762572 |
|  | *N*. *doro doro* | Luc475 | SML07 | Sun Moon Lake, Nantou County | 23.845109° | 120.924715° | AB762201 | AB762575 |

Table S1 (continued)

| **Pop** | **Taxon** | **Voucher No.** | **ID** | **Collecting Locality** | **GPS-latitude** | **GPS-longitude** | **COI** | **16S rRNA** |
| --- | --- | --- | --- | --- | --- | --- | --- | --- |
| **C** | *N*. *doro doro* | Luc478 | SML09 | Sun Moon Lake, Nantou County | 23.845109° | 120.924715° | AB762203 | AB762577 |
|  | *N*. *swinhoei* | Luc380 | ALS10 | Alishan, Chiayi County | 23.484882° | 120.717942° | AB762225 | AB762605 |
|  | *N*. *swinhoei* | Luc382 | ALS12 | Alishan, Chiayi County | 23.484882° | 120.717942° | AB762227 | AB762607 |
|  | *N*. *swinhoei* | Luc390 | ALS14 | Alishan, Chiayi County | 23.490634° | 120.753384° | AB762229 | AB762609 |
|  | *N*. *swinhoei* | Luc391 | ALS15 | Alishan, Chiayi County | 23.490634° | 120.753384° | AB762230 | AB762610 |
|  | *N*. *doro doro* | Luc1277 | ALS23 | Alishan, Chiayi County | 23.491031° | 120.721958° | LC590089 | LC590172 |
|  | *N*. *doro doro* | Luc1221 | PL09 | Dongguang Village, Nantou County | 23.874216° | 120.973031° | LC590090 | LC590173 |
|  | *N*. *swinhoei* | Luc1175 | JL01 | Sitou, Nantou County | 23.696047° | 120.747419° | LC590091 | LC590174 |
|  | *N*. *swinhoei* | Luc1212 | JL10 | Shenkeng, Nantou County | 23.696047° | 120.747419° | LC590092 | LC590175 |
|  | *N*. *swinhoei* | Luc1167 | DLS05 | Dalunshan, Nantou County | 23.681558° | 120.765967° | LC590093 | LC590176 |
|  | *N*. *swinhoei* | Luc1168 | DLS06 | Dalunshan, Nantou County | 23.681558° | 120.765967° | LC590094 | LC590177 |
|  | *N*. *swinhoei* | Luc1170 | DLS07 | Dalunshan, Nantou County | 23.681558° | 120.765967° | LC590095 | LC590178 |
|  | *N*. *swinhoei* | Luc1265 | JSH01 | Jhangshuhu, Chiayi County | 23.516960° | 120.700369° | LC590096 | LC590179 |
|  | *N*. *swinhoei* | Luc1267 | JSH03 | Jhangshuhu, Chiayi County | 23.516960° | 120.700369° | LC590097 | LC590180 |
|  | *N*. *swinhoei* | Luc1270 | JSH04 | Jhangshuhu, Chiayi County | 23.516960° | 120.700369° | LC590098 | LC590181 |
| **D** | *N*. *eugeniae* | LucAF125 | SP06 | Shanping, Kaohsiung City | 22.992011° | 120.690278° | AB762216 | AB762589 |
|  | *N*. *eugeniae* | LucAF127 | SP08 | Shanping, Kaohsiung City | 22.992011° | 120.690278° | AB762218 | AB762590 |
|  | *N*. *swinhoei* | Luc603 | SP09 | Shanping, Kaohsiung City | 22.970365° | 120.684973° | AB762249 | AB762629 |
|  | *N*. *swinhoei* | Luc604 | SP10 | Shanping, Kaohsiung City | 22.970365° | 120.684973° | AB762250 | AB762630 |
|  | *N*. *swinhoei* | Luc605 | SP11 | Shanping, Kaohsiung City | 22.970365° | 120.684973° | AB762251 | AB762631 |
|  | *N*. *swinhoei* | Luc606 | SP12 | Shanping, Kaohsiung City | 22.970365° | 120.684973° | AB762252 | AB762632 |
|  | *N*. *swinhoei* | Luc607 | SP13 | Shanping, Kaohsiung City | 22.970365° | 120.684973° | AB762253 | AB762633 |
|  | *N*. *swinhoei* | Luc618 | SP14 | Tengjhih, Kaohsiung City | 23.017584° | 120.693312° | AB762254 | AB762634 |
|  | *N*. *eugeniae* | Luc1176 | SP15 | Shanping, Kaohsiung City | 22.970365° | 120.684973° | LC590099 | LC590182 |
|  | *N*. *eugeniae* | Luc1261 | ShS01 | Shihshan, Kaohsiung City | 23.065381° | 120.760013° | LC590100 | LC590183 |
|  | *N*. *eugeniae* | Luc1262 | ShS02 | Shihshan, Kaohsiung City | 23.065381° | 120.760013° | LC590101 | LC590184 |
|  | *N*. *swinhoei* | Luc1019 | TJ01 | Tengjhih, Kaohsiung City | 23.059650° | 120.735274° | LC590102 | LC590185 |
|  | *N*. *swinhoei* | Luc1020 | TJ02 | Tengjhih, Kaohsiung City | 23.059650° | 120.735274° | LC590103 | LC590186 |
|  | *N*. *swinhoei* | Luc1021 | TJ03 | Tengjhih, Kaohsiung City | 23.059650° | 120.735274° | LC590104 | LC590187 |
|  | *N*. *swinhoei* | Luc1022 | TJ04 | Tengjhih, Kaohsiung City | 23.059650° | 120.735274° | LC590105 | LC590188 |
|  | *N*. *swinhoei* | Luc1023 | TJ05 | Tengjhih, Kaohsiung City | 23.059650° | 120.735274° | LC590106 | LC590189 |
|  | *N*. *swinhoei* | NPUST01 | WA01 | Wanan, Pingtung County | 22.625906° | 120.647321° | LC590107 | LC590190 |
|  | *N*. *swinhoei* | NPUST03 | WA02 | Wanan, Pingtung County | 22.625906° | 120.647321° | LC590108 | LC590191 |
|  | *N*. *swinhoei* | NPUST06 | WA03 | Wanan, Pingtung County | 22.625906° | 120.647321° | LC590109 | LC590192 |
|  | *N*. *swinhoei* | NPUST08 | WA04 | Wanan, Pingtung County | 22.625906° | 120.647321° | LC590110 | LC590193 |
|  | *N*. *swinhoei* | NPUST09 | WA05 | Wanan, Pingtung County | 22.625906° | 120.647321° | LC590111 | LC590194 |
|  | *N*. *swinhoei* | NPUST10 | WA06 | Wanan, Pingtung County | 22.625906° | 120.647321° | LC590112 | LC590195 |
|  | *N*. *swinhoei* | NPUST22 | WA07 | Wanan, Pingtung County | 22.625906° | 120.647321° | LC590113 | LC590196 |
|  | *N*. *swinhoei* | NPUST24 | WA08 | Wanan, Pingtung County | 22.625906° | 120.647321° | LC590114 | LC590197 |
|  | *N*. *swinhoei* | NPUST27 | WT01 | Wutai, Pingtung County | **-** | **-** | LC590115 | LC590198 |
| **E** | *N*. *swinhoei* | Luc740 | DHS01 | Dahanshan, Pingtung County | 22.413289° | 120.752743° | LC074628 | LC075129 |
|  | *N*. *swinhoei* | Luc1103 | DHS02 | Dahanshan, Pingtung County | 22.413289° | 120.752743° | LC590116 | LC590199 |
|  | *N*. *swinhoei* | Luc1163 | DHS03 | Dahanshan, Pingtung County | 22.408538° | 120.756146° | LC074645 | LC075146 |
|  | *N*. *swinhoei* | Luc1177 | DHS04 | Dahanshan, Pingtung County | 22.408538° | 120.756146° | LC074646 | LC075147 |

Table S1 (continued)

| **Pop** | **Taxon** | **Voucher No.** | **ID** | **Collecting Locality** | **GPS-latitude** | **GPS-longitude** | **COI** | **16S rRNA** |
| --- | --- | --- | --- | --- | --- | --- | --- | --- |
| **E** | *N*. *swinhoei* | Luc1178 | DHS05 | Dahanshan, Pingtung County | 22.413289° | 120.752743° | LC590117 | LC590200 |
|  | *N*. *swinhoei* | Luc1179 | DHS06 | Dahanshan, Pingtung County | 22.413289° | 120.752743° | LC590118 | LC590201 |
|  | *N*. *swinhoei* | Luc1180 | DHS07 | Dahanshan, Pingtung County | 22.413289° | 120.752743° | LC590119 | LC590202 |
|  | *N*. *swinhoei* | Luc1181 | DHS08 | Dahanshan, Pingtung County | 22.413289° | 120.752743° | LC590120 | LC590203 |
|  | *N*. *swinhoei* | Luc1182 | DHS09 | Dahanshan, Pingtung County | 22.413289° | 120.752743° | LC590121 | LC590204 |
|  | *N*. *swinhoei* | Luc1183 | DHS10 | Dahanshan, Pingtung County | 22.413289° | 120.752743° | LC590122 | LC590205 |
| **F** | *N*. *swinhoei* | Luc1078 | LS01 | Leshuei, Yilan County | 24.595437° | 121.531668° | LC074641 | LC075142 |
|  | *N*. *swinhoei* | Luc1079 | LS02 | Leshuei, Yilan County | 24.595437° | 121.531668° | LC074642 | LC075143 |
|  | *N*. *swinhoei* | Luc982 | TPS01 | Taipingshan, Yilan County | 24.534549° | 121.519133° | LC590123 | LC590206 |
|  | *N*. *swinhoei* | Luc1122 | TPS03 | Taipingshan, Yilan County | 24.534549° | 121.519133° | LC590124 | LC590207 |
|  | *N*. *swinhoei* | Luc1123 | TPS04 | Taipingshan, Yilan County | 24.534549° | 121.519133° | LC590125 | LC590208 |
|  | *N*. *swinhoei* | Luc1124 | TPS05 | Taipingshan, Yilan County | 24.534549° | 121.519133° | LC590126 | LC590209 |
|  | *N*. *swinhoei* | Luc1125 | TPS06 | Taipingshan, Yilan County | 24.534549° | 121.519133° | LC590127 | LC590210 |
|  | *N*. *swinhoei* | Luc1126 | TPS07 | Taipingshan, Yilan County | 24.534549° | 121.519133° | LC074643 | LC075144 |
|  | *N*. *swinhoei* | Luc1127 | TPS08 | Taipingshan, Yilan County | 24.534549° | 121.519133° | LC074644 | LC075145 |
|  | *N*. *swinhoei* | Luc1128 | TPS09 | Taipingshan, Yilan County | 24.534549° | 121.519133° | LC590128 | LC590211 |
| **G** | *N*. *swinhoei* | Luc1304 | SBY01 | Sinbaiyang, Hualien County | 24.186557° | 121.422158° | LC590129 | LC590212 |
|  | *N*. *swinhoei* | Luc717 | TM01 | Tongmen, Hualien County | 24.008193° | 121.395469° | LC074626 | LC075127 |
|  | *N*. *swinhoei* | Luc718 | TM02 | Tongmen, Hualien County | 24.008193° | 121.395469° | LC074627 | LC075128 |
| **H** | *N*. *swinhoei* | Luc713 | RS01 | Rueisuei, Hualien County | 23.504742° | 121.304151° | LC074623 | LC075124 |
|  | *N*. *swinhoei* | Luc714 | RS02 | Rueisuei, Hualien County | 23.504742° | 121.304151° | LC074624 | LC075125 |
|  | *N*. *swinhoei* | Luc715 | RS03 | Rueisuei, Hualien County | 23.504742° | 121.304151° | LC074625 | LC075126 |
|  | *N*. *swinhoei* | Luc1043 | RS04 | Rueisuei, Hualien County | 23.504742° | 121.304151° | LC590130 | LC590213 |
|  | *N*. *swinhoei* | Luc1045 | RS05 | Rueisuei, Hualien County | 23.504742° | 121.304151° | LC074638 | LC075139 |
|  | *N*. *swinhoei* | Luc1234 | CKS01 | Chihkeshan, Hualien County | 23.388087° | 121.391670° | LC074649 | LC075150 |
|  | *N*. *swinhoei* | Luc1235 | CKS02 | Chihkeshan, Hualien County | 23.388087° | 121.391670° | LC074650 | LC075151 |
|  | *N*. *swinhoei* | Luc1236 | CKS03 | Chihkeshan, Hualien County | 23.388087° | 121.391670° | LC074651 | LC075152 |
|  | *N*. *swinhoei* | Luc1237 | CKS04 | Chihkeshan, Hualien County | 23.388087° | 121.391670° | LC074652 | LC075153 |
|  | *N*. *swinhoei* | Luc1232 | LSDS01 | Lioushihdanshan, Hualien County | 23.224365° | 121.316732° | LC074647 | LC075148 |
|  | *N*. *swinhoei* | Luc1233 | LSDS02 | Lioushihdanshan, Hualien County | 23.224365° | 121.316732° | LC074648 | LC075149 |
| **I** | *N*. cf. *doro* | Luc609 | YP01 | Yanping, Taitung County | 22.891291° | 121.188835° | AB762266 | AB762591 |
|  | *N*. cf. *doro* | Luc610 | YP02 | Yanping, Taitung County | 22.891291° | 121.188835° | AB762267 | AB762592 |
|  | *N*. cf. *doro* | Luc612 | YP04 | Yanping, Taitung County | 22.891291° | 121.188835° | AB762269 | AB762594 |
|  | *N*. cf. *doro* | Luc617 | YP09 | Yanping, Taitung County | 22.891291° | 121.188835° | AB762274 | AB762599 |
|  | *N*. *swinhoei* | NMNS ENT_6001-3898 | LJ01 | Lijia Industry Road, Taitung County | - | - | LC590131 | LC590214 |
|  | *N*. *swinhoei* | NMNS ENT_6001-3899 | LJ02 | Lijia Industry Road, Taitung County | - | - | LC590132 | LC590215 |
|  | *N*. *swinhoei* | NMNS ENT_5101-6 | LJ03 | Lijia Industry Road, Taitung County | - | - | LC590133 | LC590216 |
|  | *N*. *swinhoei* | NMNS ENT_5101-3 | JB01 | Jhihben Trail, Taitung County | - | - | LC590134 | LC590217 |
|  | *N*. *swinhoei* | Luc1046 | JJS01 | Jinjhenshan, Taitung County | 22.627804° | 120.954337° | LC074639 | LC075140 |
|  | *N*. *swinhoei* | Luc1047 | JJS02 | Jinjhenshan, Taitung County | 22.627804° | 120.954337° | LC074640 | LC075141 |

“-”: absence data.
