# Supplementary figures and images for: Ancestral Haplotype Retention and Population Expansion Determine the Complicated Population Genetic Structure of the Hilly Lineage of Neolucanus swinhoei Complex (Coleoptera, Lucanidae) on the Subtropical Taiwan Island
Source: Insects. 2021 Mar 5;12(3):227. doi: 10.3390/insects12030227 (PMC7999642; doi:10.3390/insects12030227)

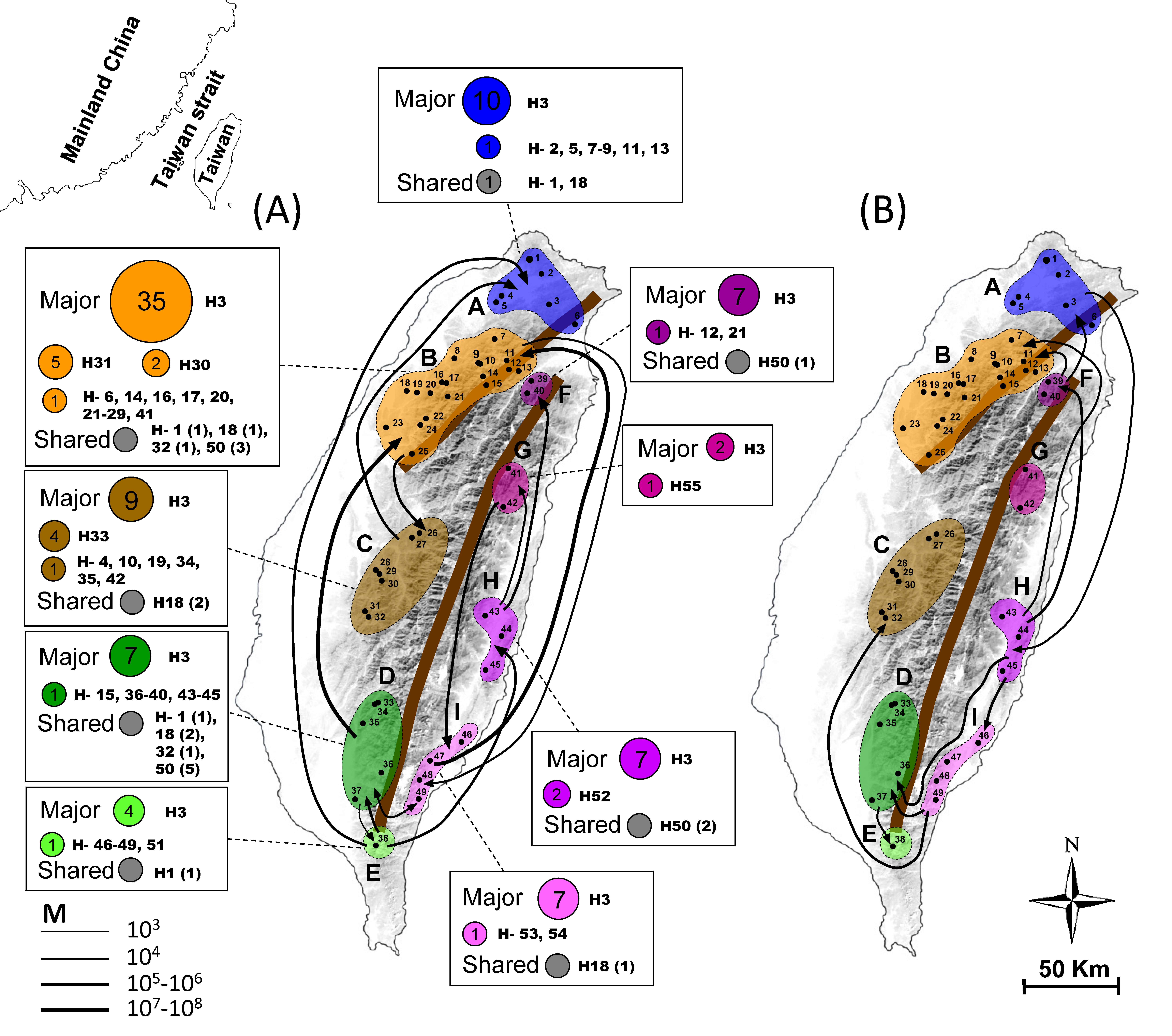

Supplement: Supplementary file 1 [file insects-12-00227-s001.zip › Figure S6.tif]
